# Ontogenetic *De Novo* Copy Number Variations (CNVs) as a Source of Genetic Individuality: Studies on Two Families with MZD Twins for Schizophrenia

**DOI:** 10.1371/journal.pone.0017125

**Published:** 2011-03-02

**Authors:** Sujit Maiti, Kiran Halagur Bhoge Gowda Kumar, Christina A. Castellani, Richard O'Reilly, Shiva M. Singh

**Affiliations:** 1 Molecular Genetics Unit, Department of Biology, The University of Western Ontario, London, Ontario, Canada; 2 Department of Psychiatry and London Health Sciences Centre, The University of Western Ontario, London, Ontario, Canada; Seattle Children's Research Institute, United States of America

## Abstract

Genetic individuality is the foundation of personalized medicine, yet its determinants are currently poorly understood. One issue is the difference between monozygotic twins that are assumed identical and have been extensively used in genetic studies for decades [1]. Here, we report genome-wide alterations in two nuclear families each with a pair of monozygotic twins discordant for schizophrenia evaluated by the Affymetrix 6.0 human SNP array. The data analysis includes characterization of copy number variations (CNVs) and single nucleotide polymorphism (SNPs). The results have identified genomic differences between twin pairs and a set of new provisional schizophrenia genes. Samples were found to have between 35 and 65 CNVs per individual. The majority of CNVs (∼80%) represented gains. In addition, ∼10% of the CNVs were *de novo* (not present in parents), of these, 30% arose during parental meiosis and 70% arose during developmental mitosis. We also observed SNPs in the twins that were absent from both parents. These constituted 0.12% of all SNPs seen in the twins. In 65% of cases these SNPs arose during meiosis compared to 35% during mitosis. The developmental mitotic origin of most CNVs that may lead to MZ twin discordance may also cause tissue differences within individuals during a single pregnancy and generate a high frequency of mosaics in the population. The results argue for enduring genome-wide changes during cellular transmission, often ignored in most genetic analyses.

## Introduction

Genome-wide constancy and change underlies evolution and familial inheritance but remains ill-defined. An assessment of changes as the genome is passed on from one generation (meiosis) and developmental cycle (mitosis) to the next is needed. It directly contributes to the sum of genetic individuality. At present, these inquiries are difficult [2], and require the development of new quantitative methods to assess genome-wide changes and their significance. This report assesses two common measures of genomic variation: copy number variations and (CNVs) and single nucleotide polymorphism (SNPs) across a generation and between monozygotic twins in two exceptional families. The results offer novel insight into meiotic and mitotic sources of variation, which results in genetic individuality between MZ twins. This individuality may account for discordance in monozygotic twins for a variety of diseases including schizophrenia.

CNVs are structural variants that are both frequent and relevant and may range in size in humans from 1 Kb to several Mb [3]. Given their impact on physiology and function, CNVs have a major influence on evolution and gene expression and on normal and disease related variation [3]. CNVs include duplications and deletions leading to a departure from the classic view that all autosomal genes are present in two copies, with one allele inherited from each parent. The majority of CNVs are copy number polymorphisms (CNPs), existing in a frequency that is greater than 1% and transmitted across generations. However, a small proportion of CNVs are novel events. CNVs may account for a major fraction (∼12%) of the genome, but appear to concentrate in some genomic regions depending on the sequence features [4], [5]. Unlike CNVs, SNPs are relatively small changes, usually involving replacement of a nucleotide with another. SNPs are common and distributed across the entire human genome. Individual SNPs mark a unique genomic location, and are usually neutral in nature. In other cases, they may change amino-acids, cause protein truncation or affect expression. They are easily detected, and have been extensively exploited in genetic analysis including the cloning of disease causing genes, individual identification and establishment of genetic relatedness.

**Figure 1:**
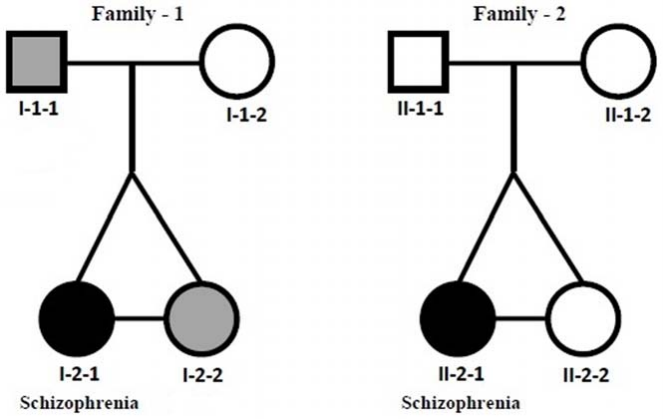
Pedigree of two families with monozygotic twins discordant for schizophrenia. Members of the family one are indicated with (I-) and members of the family two are indicated with (II-). The designations included in this figure are followed in subsequent figures and tables.

Studies on these two genome-wide variations (SNPs and CNVs) have greatly enhanced our understanding of evolution and genetic individuality. They are also helping to elucidate the cause of genetic, and genomic disorders including schizophrenia [6]. A number of SNPs appear to be linked to this complex neuro-developmental disease, which has a heritability estimate of 80%. However, results of linkage studies have not been consistently reproducible [7], [8]. Individuals affected with schizophrenia (SCZ) have shown an elevated incidence of CNVs [9] and a few rare CNVs appear to have a major effect on the development of SCZ [10]. However, these CNVs account for only a small fraction of schizophrenia cases [11] and the challenge of identifying common genetic cause(s) of SCZ remains. The search for genes in SCZ currently relies on large number of patients and matched controls. The limited progress using these approaches emphasizes the need to pursue alternative approaches. Future studies may benefit from inclusion of two features. The first is a genome-wide comparison of the parents and their progeny affected by SCZ and the second is the assessment of genomes of monozygotic twins (that show ∼52% discordance for SCZ) [12], [13]. The current study reports genome-wide CNV and SNP results on two exceptional families that include monozygotic twins discordant for schizophrenia (Figure 1, Table 1).

**Table 1:** Demography and Clinical History.

Family 1					Family 2			
	I-1-1	I-1-2	I-2-1	I-2-2	II-1-1	II-1-2	II-2-1	II-2-2
**Age (yr.) at assessment**	82	74	53	53	N/A	N/A	43	43
**Sex**	Male	Female	Female	Female	Male	Female	Female	Female
**Declared Race**	Afro-American				Caucasian			
**Psychiatric features**	Compulsive Personality Disorder	N/A	Schizophrenia, Paranoid Type, onset age 22	Bipolar I Disorder, onset age 52	Major depression and panic disorder for 6 months after cardiac surgery, onset age 73	N/A	Schizoaffective Disorder, onset age 27	Single episode of Major Depression, fully remitted, onset age 18

Demography and Clinical History of monozygotic (MZ) twins discordant for Schizophrenia (SCZ). Family one is indicated with (I), family two is indicated with (II). N/A = Not Applicable.

## Results and Discussion

### Familial Distribution of CNVs

**Figure 2:**
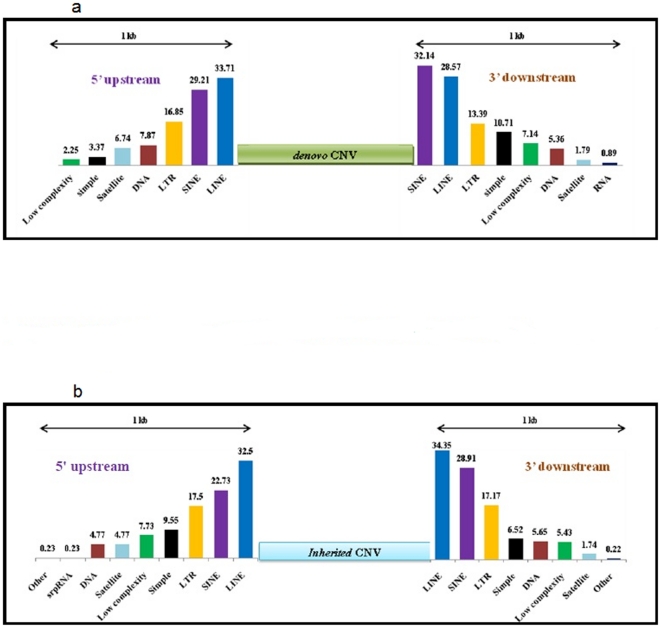
Distribution of repeat elements 1 kb upstream (5′) and 1 kb downstream (3′) of the *de novo* (2a) and *inherited* (2b) CNVs across eight individuals. These include LINE (blue), SINE (purple), LTR (yellow), Satellite (sky blue), simple repeats (black) and low complexity repeats (green) with numerical values on top of the bars representing percentage of that repeat.

The number of CNVs per individual ranged from 35 to 65, with the exception of one individual who is described more fully later (Table 2). This is similar to the number of CNVs per subject reported from most other studies that have used Affymetrix 6.0 Human SNP arrays [14]. The range is also comparable with the number of CNVs found in Venter's genome (62) based on his complete genome sequence [15]. The exception in our study was the father in family 2 (II-1-1) who was found to harbour a rare chromosome 13q deletion containing 40 CNVs at a single genomic location. Although this finding is beyond the scope of this report, it is important to note that II-1-1 underwent chemotherapy treatment and that the samples utilized in this study were obtained towards the end of that treatment. Most CNVs identified were in the range of 100 to 200 Kb, consistent with the size distribution of CNVs reported in the literature [14]. The majority of CNVs observed (Table 3) were copy number gains (78.5%) and ∼10% of the CNVs identified are not listed in the Database of Genomic Variants (http://projects.tcag.ca/variation/) accessed on 8.2.2010. Further, the chromosomal distribution of CNVs was comparable across individuals with the exception of the father in family 2 who had consistently higher CNVs affecting most chromosomes (Table 4). Of the CNVs identified, >50 per cent overlapped RefSeq genes. The identified genes are frequently associated with metabolic pathways such as starch and sucrose metabolism as well as pathways involved in the metabolism of amino acids, for example, , phenylalanine, histidine and tyrosine (*AMY2A,AMY1A,ALDH1L1,PSMC1*). Structurally, >67% of the CNVs identified were flanked at both the 5′and 3′ end or at just the 5′ (>7%) or 3′ (>8%) end with a set of common repeats, represented by short interspersed nucleotide elements (SINEs), long interspersed nucleotide elements (LINEs), long terminal repeats (LTRs) and low copy repeats (LCRs) near the breakpoints. The majority of the deletion breakpoints had 1–30 bp of microhomology, whereas a small fraction of deletion breakpoints contained inserted sequences. The co-occurrence of microhomology and inserted sequence suggests that both recombination and replication based mutational mechanisms are operational in CNV generation. Recent studies have identified short DNA motifs that both determine the location of meiotic crossover hotspots and are significantly enriched at the breakpoints of recurrent non-allelic homologous recombination (NAHR) syndromes [16]. We found evidence for this mechanism in a subset of the breakpoint events (data not shown). This was true for the *de novo* (Figure 2a) as well as inherited (Figure 2b) CNVs. Such sequences may represent genomic architecture that is prone to genome instability by a predisposition to genomic rearrangements via non-homologous end joining (NHEJ), template switching and/or non-allelic homologous recombination (NAHR).

**Table 2:** Distribution of CNV among family members according to size.

CNV Size	Family 1				Family 2			
	I-1-1	I-1-2	I-2-1	I-2-2	II-1-1	II-1-2	II-2-1	II-2-2
**< = 100 kb**	0	0	0	0	2	0	0	1
**>100 to 200 kb**	17	18	15	20	119	50	24	24
**>200 to 300 kb**	11	6	4	10	25	6	13	9
**>300 to 400 kb**	5	5	6	5	11	3	1	4
**>400 to 500 kb**	2	0	2	2	6	1	1	2
**>500 to 1000 kb**	6	2	4	7	7	2	5	2
**>1 to 10 Mb**	9	4	5	3	5	2	4	5
**>10 to 20 Mb**	5	0	0	0	0	0	0	0
**>20 Mb**	3	0	0	0	2	0	2	2
**Total**	**58**	**35**	**36**	**47**	**177**	**64**	**50**	**49**

Numerical values in each cell of the table indicate how many CNVs of that particular size range were observed in that particular individual.

**Table 3 pone-0017125-t003:** Identity of copy number variants across individual family members.

CNVs	Family 1				Family 2			
	I-1-1	I-1-2	I-2-1	I-2-2	II-1-1	II-1-2	II-2-1	II-2-2
**No. of Loss**	21	6	5	6	52	11	6	4
**No. of Gain**	37	29	31	41	125	53	44	45
**Novel (absent in DGV)**	1	1	0	2	42	6	1	0
**Present in DGV**	57	34	36	45	135	58	49	49
**Total (for the individual)**	**58**	**35**	**36**	**47**	**177**	**64**	**50**	**49**

Frequency of CNVs which are losses (deletion) or gains (duplication) and characterization as present or absent from The Database of Genomic Variants (DGV).

**Table 4:** Chromosome wise distribution of CNV.

Chr No.	Family 1				Family 2			
	I-1-1	I-1-2	I-2-1	I-2-2	II-1-1	II-1-2	II-2-1	II-2-2
**1**	4	2	2	2	11	2	6	6
**2**	4	2	5	5	11	2	2	3
**3**	1	4	2	3	7	4	4	2
**4**	4	3	2	4	8	1	3	2
**5**	0	0	0	0	12	0	2	1
**6**	0	0	0	0	10	2	0	0
**7**	2	6	3	4	10	5	3	4
**8**	1	0	3	3	7	3	3	3
**9**	1	1	2	3	4	4	3	4
**10**	2	1	1	1	3	5	1	1
**11**	3	1	2	1	5	1	2	2
**12**	0	1	0	1	4	3	0	1
**13**	0	0	0	0	40	1	1	0
**14**	4	6	3	5	4	6	5	3
**15**	4	3	1	3	2	6	6	8
**16**	2	1	2	3	9	1	1	1
**17**	4	1	2	3	8	2	2	1
**18**	0	0	0	0	1	0	0	1
**19**	0	1	0	0	7	3	2	1
**20**	0	0	0	0	0	0	0	1
**21**	1	2	2	3	2	3	2	1
**22**	3	0	3	2	3	1	1	1
**X**	18	0	1	1	9	9	1	2
**Total**	**58**	**35**	**36**	**47**	**177**	**64**	**50**	**49**

Chromosome specific distribution of *de novo* (present in twin(s) and not in parents) and *inherited* (present in at least one parent) CNVs in family 1 and family 2.

Chr. No = Chromosome number.

### Familial vs *de novo* Origin of CNVs

A novel feature of the data included in this report is that we are able to classify observed CNVs into two groups based on their absence or presence in one of the parents. CNVs that were found in one or both twins and not seen in either parent, were classified as *de novo*. If a *de novo* CNV was present in both twins, it was considered to have originated during parental meiosis and when present in only one of the two twins, it was assumed to have originated in mitosis during development. This classification allowed us to identify 14 and 26 *de novo* CNVs in family 1 (Table 5) and family 2 (Table 6) respectively. The table includes genomic locations as well as individual specific break points which allow for the assessment of regions of overlap with the Database of Genomic Variants (Toronto, Ontario). Mitotic origin of CNVs was ∼3 times higher than CNVs generated during parental meiosis. Of the mitotic *de novo* CNVs identified two (loss at 14q32.11 as well as loss at 8q11.21) were specific to the schizophrenia patient in family 1 and one (gain at 19q13.41) was specific to the patient in family 2. Such results are novel in the literature. Further, it is enticing to ask the question, do the genes disturbed by CNVs contribute to the development of their disease symptoms? Although the answers to such questions are of paramount importance, the results available do not offer a direct assessment of such questions. Nonetheless, it is appropriate to entertain the discussion that the known features of these genes are or are not compatible with disturbances observed in schizophrenia, which is discussed below.

**Table 5:** *de novo* CNVs in Family 1.

Sl. No	Location	Family 1						Status	Meiosis	Mitosis	Novel	Genes (Overlapping or Nearby)	SD
		I-2-1	Size (kb)	Breakpoints	I-2-2	Size (kb)	Breakpoints						
1	**1p36.13**	Yes	112	16724089…16835888				**Gain**		**Yes**		NBPF1, NBPF10	1
2	**2p25.3**	Yes	152	1407209…1559511	Yes	152	1407209…1559511	**Gain**	**Yes**			TPO	0
3	**2p11.2**	Yes	1147	89862331…91008912	Yes	1159	89850279…91008912	**Loss**	**Yes**				0
4	**4q28.3**				Yes	191	132801221…132992517	**Gain**		**Yes**			1
5	**7q11.21**	Yes	118	64706066…64823721	Yes	118	64704377…64822216	**Loss**	**Yes**				1
6	**8p23.1**	Yes	126	7847289…7973253				**Loss**		**Yes**			1
7	**8q11.1**	Yes	336	47045602…47381308	Yes	250	47131383…47381308	**Gain**	**Yes**				0
8	**8q11.21**				Yes	154	48178242…48332398	**Loss**		**Yes**	**Yes**	**KIAA0146**	0
9	**9p11.2**	Yes	569	45361389…45929992				**Gain**		**Yes**		FAM27A	1
10	**9p13.1**				Yes	141	38777481…38918566	**Gain**		**Yes**			1
11	**9q12**				Yes	861	65412415…66273526	**Gain**		**Yes**			1
12	**12p13.31**				Yes	196	8303317…8499801	**Gain**		**Yes**		CLEC6A	1
13	**14q32.11**				Yes	103	89780137…89883415	**Loss**		**Yes**	**Yes**	**PSMC1, C14orf102**	0
14	**21q11.2**				Yes	119	13891136…14009908	**Gain**		**Yes**		ANKRD21, LOC441956	1
15	**Xp11.23**	Yes	149	47917899…48066856	Yes	149	47917899…48066856	**Gain**	**Yes**			SSX5, SSX1, SSX9	0

**Table 6 pone-0017125-t006:** *de novo* CNVs in Family 2.

Sl. No	Location	Family 2						Status	Meiosis	Mitosis	Novel	Genes (Overlapping or Nearby)	SD
		II-2-1	Size (kb)	Breakpoints	II-2-2	Size (kb)	Breakpoints						
1	**1q21.1**				Yes	120	143867807…143987616	**Gain**		**Yes**		NOTCH2NL	0
2	**1q21.1**	Yes	104	147353175…147456930	Yes	104	147353175…147456930	**Loss**	**Yes**				0
3	**1q43**				Yes	119	241230453…241349107	**Gain**		**Yes**			0
4	**3q21.2**	Yes	155	126958012…127112518				**Gain**		**Yes**			1
5	**4p11**	Yes	299	48986100…49285347				**Gain**		**Yes**			0
6	**5p15.33**	Yes	101	770367…871743	Yes	107	770367…877436	**Gain**	**Yes**			ZDHHC11	1
7	**5p13.3**	Yes	151	34119387…34269887				**Gain**		**Yes**			1
8	**7q11.21**	Yes	202	61761008…61962936				**Gain**		**Yes**			0
9	**7q11.21**	Yes	116	64588316…64704125	Yes	125	64579322…64704125	**Gain**	**Yes**				1
10	**7q35**				Yes	100	142956516…143056637	**Gain**		**Yes**		LOC441294, FAM139A	1
11	**8p23.1**	Yes	220	12071704…12291845	Yes	220	12071704…12291845	**Gain**	**Yes**			FAM86B1, DEFB130	0
12	**9p12**	Yes	2720	41465094…44184864	Yes	1901	42249132…44149779	**Gain**	**Yes**			ANKRD20A2, ANKRD20A3, FOXD4L4, FOXD4L2	1
13	**9q12**				Yes	250	67416254…67665974	**Gain**		**Yes**		ANKRD20A1, ANKRD20A3	1
14	**11q13.2**	Yes	267	67239223…67505822	Yes	139	67239223…67378031	**Gain**	**Yes**				1
15	**12p13.31**				Yes	189	8310909…8499801	**Gain**		**Yes**			1
16	**13q11**	Yes	208	18138676…18346383				**Gain**		**Yes**			1
17	**14q11.1**	Yes	601	18072112…18672662	Yes	601	18072112…18672662	**Gain**	**Yes**			OR11H12, ACTBL1	1
18	**15q11.1**				Yes	106	18276329…18382609	**Gain**		**Yes**			1
19	**15q11.2**	Yes	203	19882763…20085783	Yes	221	19864583…20085783	**Gain**	**Yes**			OR4M2, OR4N4, LOC650137	1
20	**15q13.1**				Yes	227	26808083…27035216	**Gain**		**Yes**		APBA2	0
21	**15q13.2**	Yes	110	28452853…28563274				**Gain**		**Yes**		CHRFAM7A	1
22	**17p11.1**	Yes	199	22127012…22326425				**Gain**		**Yes**			0
23	**19q13.41**	Yes	109	58847652…58957090				**Gain**		**Yes**	**Yes**	**ZNF331, DPRX**	0
24	**20q11.1**				Yes	118	28147331…28264860	**Gain**		**Yes**			1
25	**21p11.2**	Yes	3480	10106540…13586186	Yes	3814	9758730…13572586	**Gain**	**Yes**			BAGE2, BAGE4, BAGE	0

Identity of *de novo* CNVs found in Family 1 (5a) and Family 2 (5b) and the gene regions (overlapping or nearby). *De novo* CNVs are defined as those that are present in either or both twins but not found in parents. SD displays the percentage of overlap with segmental duplications, ‘0’ indicates no overlap between the CNV and segmental duplication and ‘1’ indicates 90–100% overlap. The table includes genomic locations as well as twin specific breakpoints which allow for the assessment of regions of overlap with the Database of Genomic Variants (Toronto, Ontario). SI No. = Serial number. Novel indicates a CNV which is not present in The Database of Genomic Variants (DGV).

### *De novo* CNVs and Schizophrenia

The genes overlapping disease specific *de novo* CNVs in family 1 included *PSMC1* (proteasome 26S subunit, ATPase, 1) and C14orf102 (chromosome 14 open reading frame 102 gene) on 14q32.11 and *KIAA0146* on 8q11.21. *PSMC1* (MIM 602706) is an ATP-dependent protease [17] that may include protein ubiquitination in response to DNA damage [18]. It is composed of a 20S catalytic proteasome and 2 PA700 regulatory modules and contains an AAA (ATPases associated with diverse cellular activities) domain [17]. The human and mouse proteins are 99% identical [19] and may play a significant role in ubiquitin-mediated proteasomal proteolysis in the molecular pathogenesis of neurological diseases such as spinocerebellar ataxia type 7 (SCA7). Also, several studies (for review, see [20], [21]), have indicated that the genes related to ubiquitination are altered in the brains of patients with schizophrenia. Further, this CNV also affects another gene (*C14orf102*; chromosome 14 open reading frame 102) which is conserved across phyla and highly expressed in the brain (Affymetrix GNF Expression Atlas 2 Data). The other CNV affected in this patient of family 1 represents a loss at 8q11.21, that contains the still uncharacterized gene, *KIAA0146*, which is expressed in the brain, may contain a CAG repeat and is conserved in chimpanzee, dog, cow, mouse, rat, chicken, and zebra fish. It is a transcription factor with CCAAT enhancer binding protein (CEBP) function [22]. Further the gene is highly expressed in the brain and hippocampus that may implicate it in mental disorders (www.genecards.org). Although we cannot rule out a role for these three genes (*PSMC1*, *C14orf102* and *KIAA0146*) in schizophrenia, such conclusions would be premature. Only a follow up study will establish if any of the three genes directly contribute to the development of schizophrenia in the patient from family 1. A similar analysis of CNVs in family 2 has identified a 109 kb gain at 19q13.41 that is specific to the schizophrenia patient in family 2. Translocations involving 19q13 are a frequent finding in follicular adenomas of the thyroid and may represent the most frequent type of structural aberration in human epithelial tumors [23]. The CNV identified in this region contains two genes; *DPRX1* and *ZNF331*. *DPRX1* (divergent-paired related homeobox) is a member of the *DPRX* homeobox gene family, contains a single conserved homeodomain and may function as a putative transcription factor. It may bind a promoter or enhancer sequence or interact with a DNA binding transcription factor and is involved in early embryonic development and cell differentiation [24]. The drosophila homologue of the *DPRX1* gene (dPrx5; Drosophila peroxiredoxin 5) confers protection against oxidative stress, apoptosis and also promotes longevity [25]. The next gene, *ZNF331*(zinc finger protein 331) affected by this CNV is also involved in DNA-dependent regulation of transcription as a transcriptional repressor [26]. Interestingly, it is one of the imprinted genes that exhibits monoallelic expression in a parent-of-origin specific manner [27]. Imprinted genes are important for development and behaviour and disruption of their expression is associated with many human disorders [28]. In conclusion the three genes affected in the schizophrenia patient in family 1 (*PSMC1*, *C14orf102*, *KIAA0146*) and the two genes affected in the patient of family 2 (*DPRX1 and ZNF331*) could not be excluded from their potential involvement in the development of schizophrenia in the two patients. If applicable, the biological systems affected in the two patients is hypothesized to be different. The patient in family one is hypothesized to have a ubiquitin-mediated proteasomal proteolysis while the patient of family 2 could have errors in regulatory mechanisms affecting gene regulation. Such conclusions must remain hypothetical until proven by independent supporting evidence.

### *De novo* changes may lead to mosaicism

The genotypes generated by the Affymetrix 6.0 array have also allowed us to establish that ∼0.12% (1086 and 1022 in twin pair 1 and 2 respectively; 11 substitutions shared by both pairs) of the SNPs in the twins represented *de novo* substitutions, but unlike CNVs, (that primarily originated during ontogeny in mitosis) most (63–65%) originated during parental meiosis. These results suggest that DNA replication fidelity at the level of single base pairs (SNPs) vs replication forks (CNVs) is differentially exercised during meiosis and mitosis. The single base pairing is much more stringent in mitosis (evolved to produce identical daughter cells), compared to meiosis where errors can facilitate potentially beneficial variations. In contrast, CNVs which affect the phenotype may be advantageous when occurring during mitosis and selected for during development. Thus, cell type specific CNVs may play a role in growth and development, offering advantageous variability. This would mean that most individuals are mosaics [29]: a hypothesis that is difficult to assess and evaluate. It is likely that the ratio of mosaic cells may be maintained throughout the differentiated (ectoderm, mesoderm, endoderm, etc) tissues over the lifetime [30], [31]; an exception being when other factors are directly influencing DNA stability. Such a mechanism may generate genomic differences and differential mosaicism in most or all individuals. If this is the case, it will complicate traditional genetic analysis that assumes stability of the genome with rare exceptions.

We have been able to establish genome-wide (CNVs and SNPs) discordance for MZ twin pairs. Also, given that the twins are discordant for schizophrenia, it is possible to assign provisional CNVs (and genes) as well as substitutions (SNPs) that may be associated with the disease status of the affected twins in family 1 and family 2 (Table 7,8). Similarly, we identified substitutions (SNPs) that were different between the affected and unaffected member of the two sets of twins including their distribution along the chromosomes, introns and exons and the predicted effect on the gene product. Identity of *de novo* CNVs found in Family 1 (Table 5) and Family 2 (Table 6) and the gene regions which they overlap was reported. *De novo* CNVs are defined as those that are present in either twin but not found in parents. In the tables, SD indicates the percentage of overlap between segmental duplications and the CNVs, ‘0’ means there is no overlap between CNV and segmental duplication and ‘1’ means 90–100% overlap.

**Table 7 pone-0017125-t007:** Inherited CNVs in Family 1.

Sl. No	Location	Family 1												Status	Novel	Genes (Overlapping or Nearby)	SD
		I-1-1	Size(kb)	Breakpoints	I-1-2	Size(kb)	Breakpoints	I-2-1	Size(kb)	Breakpoints	I-2-2	Size(kb)	Breakpoints				
1	1p36.33	Yes	167	51586…218557				Yes	707	51586…758644	Yes	707	51586…758644	**Gain**		OR4F5, OR4F3, OR4F16, OR4F29	1
2	1q21.1	Yes	765	147303136…148068045	Yes	734	147311699…148045353				Yes	577	147381253…147958358	**Gain**		PPIAL4, FCGR1A, HIST2H2BF	1
3	2p11.2	Yes	338	88917155…89254935	Yes	322	88914734…89236978	Yes	325	88917155…89242149	Yes	327	88914734…89242149	**Gain**			1
4	2p11.1				Yes	138	91017077…91154841	Yes	143	91017077…91160399	Yes	137	91017077…91154463	**Gain**			1
5	2q21.2	Yes	236	132597824…132833718				Yes	222	132597824…132819911	Yes	222	132597824…132819911	**Gain**			1
6	3p12.3	Yes	306	75677859…75984129	Yes	380	75597086…75977210	Yes	182	75583442…75764996	Yes	402	75582277…75984129	**Gain**			1
7	3q21.2				Yes	132	126907150…127039328				Yes	185	126907150…127091652	**Gain**			1
8	3q21.3				Yes	170	131198515…131368353	Yes	166	131213377…131379054	Yes	176	131214431…131389948	**Gain**			1
9	4p16.2				Yes	196	4040542…4236511	Yes	363	3873500…4236511	Yes	366	3870638…4236511	**Gain**			0
10	4p11	Yes	436	48849363…49285347				Yes	497	48788531…49285347	Yes	497	48788531…49285347	**Gain**			1
11	4q35.2	Yes	232	191021837…191254119	Yes	195	191059369…191254119				Yes	223	191031042…191254119	**Gain**		FRG1, TUBB4Q, FRG2, DUX4	0
12	7p11.1	Yes	231	57523223…57753919	Yes	101	57640100…57741512	Yes	315	57640100…57954861	Yes	117	57640100…57757406	**Gain**			1
13	7q11.21				Yes	112	61365830…61477958				Yes	111	61365830…61476918	**Gain**			0
14	7q11.21				Yes	253	64320173…64573380	Yes	415	64204380…64619667	Yes	385	64204380…64589253	**Loss**		ZNF92	0
15	8p23.1	Yes	694	7209579…7903560				Yes	215	7027251…7242508	Yes	270	7021193…7291135	**Loss**		DEFB103A, DEFB103B, SPAG11B, DEFB104B, DEFB104A, DEFB106B, DEFB106A, DEFB105B, DEFB105A, DEFB107B, DEFB107A, SPAG11A, DEFB4	2
16	9q12				Yes	690	68115006…68805366	Yes	1141	68115006…69256300	Yes	694	68115006…68809437	**Gain**		FOXD4L6, CBWD6, ANKRD20A4, CCDC29	1
17	10q11.1	Yes	183	41972779…42155347	Yes	105	41934430…42039743	Yes	183	41972779…42155347	Yes	239	41934430…42173117	**Gain**			1
18	11p15.4	Yes	175	3405799…3580813	Yes	131	3430789…3561991	Yes	139	3430789…3569305	Yes	156	3406002…3561991	**Gain**			1
19	11q13.2	Yes	227	67239223…67466368				Yes	193	67273413…67466368				**Gain**			1
20	14q11.1	Yes	1322	18138794…19460382	Yes	1103	18072112…19175240	Yes	705	18072112…18776746	Yes	705	18072112…18776746	**Gain**		OR11H12, ACTBL1, OR4Q3, OR4M1, OR4N2, OR4K5	0
21	14q32.33	Yes	126	105265510…105391419	Yes	167	105100670…105268160	Yes	632	105190672…105822317	Yes	181	105149735…105331052	**Gain**			0
		Yes	156	105413825…105569826	Yes	213	105289618…105502685	Yes	178	105827891…106005581	Yes	261	105341035…105601720	**Gain**			0
		Yes	205	105612786…105818132	Yes	279	105508896…105788389				Yes	280	105612786…105892769	**Gain**			0
22	15q11.1	Yes	471	18370252…18841457	Yes	178	18522238…18700540	Yes	1223	18845990…20068512	Yes	562	18276329…18838423	**Gain**		LOC283755, POTE15, OR4M2	1
		Yes	1177	18845990…20022565	Yes	344	18845990…19189673				Yes	1078	18845990…19923712	**Gain**		OR4N4, LOC650137	1
					Yes	264	19303160…19566863							**Gain**			1
23	15q11.2	Yes	189	22026287…22214843							Yes	174	22026287…22200408	**Gain**			1
24	16p11.2	Yes	1217	32303108…33520394	Yes	1297	32538757…33836128	Yes	1142	32538757…33680554	Yes	249	32538757…32787273	**Gain**		LOC729355, TP53TG3	1
											Yes	752	32910319…33662480	**Gain**			1
25	16p11.2	Yes	250	34374795…34624994				Yes	249	34375533…34624994	Yes	249	34375533…34624994	**Gain**			1
26	17p11.2	Yes	140	20559979…20700133							Yes	164	20538867…20703365	**Gain**			1
27	17q21.31	Yes	229	41521621…41750183				Yes	123	41521621…41644356	Yes	123	41521621…41644356	**Gain**		KIAA1267, LRRC37A	0
28	17q21.31	Yes	351	41756820…42107467	Yes	296	41811739…42107467	Yes	392	41700624…42092926	Yes	302	41700624…42002447	**Gain/Loss**		ARL17, LRRC37A2, NSF	1
29	21p11.2				Yes	204	9758730…9962501	Yes	204	9758730…9962501	Yes	204	9758730…9962501	**Gain**		TPTE	0
30	21p11.1	Yes	3411	10106540…13517603	Yes	3419	10106540…13525448	Yes	3419	10106540…13525448	Yes	3477	10106540…13583117	**Gain**		BAGE2, BAGE4, BAGE	2
31	22q11.1	Yes	339	14435171…14774593				Yes	320	14435207…14754960	Yes	320	14435207…14754960	**Gain**		ACTBL1	1
32	22q11.21	Yes	124	20051708…20175282				Yes	136	20145854…20281562	Yes	136	20145854…20281562	**Gain**		HIC2, UBE2L3	1
33	22q11.22	Yes	240	21292462…21532509				Yes	127	21327799…21454509				**Gain**		GGTL4	0

**Table 8:** Inherited CNVs in Family 2.

Sl. No	Location	Family 2												Status	Novel	Genes (Overlapping or Nearby)	SD
		II-1-1	Size(kb)	Breakpoints	II-1-2	Size(kb)	Breakpoints	II-2-1	Size(kb)	Breakpoints	II-2-2	Size(kb)	Breakpoints				
1	1p36.33	Yes	707	51586…758644				Yes	537	218557…755132				**Gain**		OR4F5, OR4F3, OR4F16, OR4F29	1
2	1p36.13	Yes	117	16718622…16835888				Yes	167	16718622…16885360	Yes	345	16718622…17063437	**Gain**		NBPF1, NBPF10	1
3	1p21.1				Yes	130	103910749…104041200	Yes	127	103931691…104058426				**Gain**		AMY2B, AMY2A, AMY1A, AMY1C, AMY1B	1
4	1p11.2	Yes	21680	121045307…142725034				Yes	21725	121045307…142770353	Yes	21725	121045307…142770353	**Gain**			0
5	1q23.3	Yes	121	159775403…159896554				Yes	116	159780383…159896554	Yes	121	159775403…159896554	**Loss**		FCGR3A, FCGR2C, FCGR3B	1
6	2p11.2	Yes	401	88925215…89326446	Yes	759	88914227…89673147	Yes	935	88926972…89861763	Yes	450	88914734…89365010	**Gain**			1
7	2p11.1	Yes	160	91017077…91176948				Yes	268	91017077…91285520	Yes	1275	89879561…91154463	**Gain**			1
8	2q21.2	Yes	260	132593436…132853218	Yes	183	132597824…132780848				Yes	222	132597824…132819911	**Gain**			1
9	3p12.3	Yes	260	75583442…75843060	Yes	226	75538978…75764996	Yes	260	75583442…75843060	Yes	182	75583442…75764996	**Gain**			1
10	3q12.2	Yes	108	101822746…101930873				Yes	102	101822746…101925168				**Gain**		GPR128, TFG	0
11	3q21.3				Yes	141	131198817…131339424	Yes	195	131194669…131389948	Yes	199	131198515…131397648	**Gain**			1
12	4p16.2	Yes	407	3870638…4278016	Yes	180	3964803…4144453	Yes	366	3870638…4236511	Yes	357	3870638…4227503	**Gain**		OTOP1	0
13	4q35.2	Yes	200	191053845…191254119				Yes	226	191028537…191254119	Yes	158	191052245…191210542	**Gain**		FRG1, TUBB4Q, FRG2, DUX4	0
14	7p22.1	Yes	157	6838697…6995298							Yes	155	6840798…6995298	**Gain**			1
15	7p11.1	Yes	117	57640100…57757406	Yes	114	57640100…57753919	Yes	149	57604989…57753919	Yes	123	57597399…57720623	**Gain**			1
16	8p23.1	Yes	203	12415742…12618442				Yes	199	12415742…12614748	Yes	136	12415742…12551430	**Gain**			1
17	8p11.23	Yes	139	39349470…39488053	Yes	151	39354748…39506110	Yes	133	39354748…39488053	Yes	151	39354748…39506110	**Loss**			0
18	9p11.2	Yes	21937	44336683…66273526				Yes	21015	45258754…66273526	Yes	21015	45258754…66273526	**Gain**		FAM27A, FAM75A7	0
	9q12	Yes	861	68352238…69213455	Yes	103	68115006…68218485	Yes	1180	68076544…69256300	Yes	1099	68115006…69213671	**Gain**		FOXD4L6, CBWD6, ANKRD20A4, CCDC29	1
					Yes	457	68352238…68809437							**Gain**			
19	10q11.1	Yes	129	41974796…42103488	Yes	236	41934430…42170853	Yes	105	41934430…42039743	Yes	239	41934430…42173117	**Gain**			1
20	11p15.4	Yes	206	3383178…3588946				Yes	205	3376078…3580813	Yes	131	3430789…3561991	**Gain**			1
21	14q11.2				Yes	186	21602854…21788783	Yes	172	21625813…21787161				**Loss**			0
22	14q11.2				Yes	226	21804698…22030660	Yes	226	21804698…22030660				**Loss**			0
23	14q32.33	Yes	386	105190672…105576359	Yes	253	105345270…105597999	Yes	411	105190672…105601397	Yes	336	105265510…105601397	**Gain**			0
24	14q32.33	Yes	137	105760582…105897672	Yes	173	105645593…105818132	Yes	150	105638133…105788389	Yes	182	105640496…105822317	**Gain**			0
25	15q11.2				Yes	156	18682380…18838423	Yes	183	18655531…18838423	Yes	156	18682380…18838423	**Gain**		LOC283755, POTE15	1
					Yes	1067	18861808…19928521	Yes	572	18850029…19422452	Yes	344	18845990…19189673	**Gain**		OR4M2, OR4N4, LOC650137	1
											Yes	624	19207088…19835514	**Gain**			1
26	15q25.3				Yes	161	83524791…83685356	Yes	228	83524791…83752853	Yes	228	83524791…83752450	**Gain**		AKAP12	0
27	15q25.3				Yes	123	83784507…83907801	Yes	157	83784507…83941483	Yes	159	83790259…83949305	**Gain**		AKAP13	0
28	16p11.2	Yes	118	31882658…32000323				Yes	1131	32531735…33662480	Yes	1377	32303108…33680554	**Gain**		LOC729355, TP53TG3	1
		Yes	294	32088275…32382422										**Gain**			1
		Yes	185	32538757…32723310										**Gain**			1
		Yes	474	32962147…33436245										**Gain**			1
		Yes	211	33451476…33662480										**Gain**			1
29	17q21.31	Yes	586	41521621…42107467	Yes	198	41521621…41719935	Yes	586	41521621…42107467	Yes	407	41700624…42107467	**Gain**		KIAA1267, LRRC37A, ARL17, LRRC37A2, NSF	1
30	18p11.21	Yes	1545	15262486…16807594							Yes	130	15218647…15348836	**Gain**		ROCK1	1
31	19q13.31	Yes	116	47991257…48107552				Yes	133	47991257…48123857	Yes	235	47986218…48221228	**Loss**		PSG1, PSG6, PSG7, PSG11	1
32	21p11.2	Yes	204	9758730…9962501	Yes	204	9758730…9962501	Yes	204	9758730…9962501				**Gain**		TPTE	0
33	22q11.22	Yes	148	21300127…21448190				Yes	200	21298324…21498767	Yes	178	21298324…21476564	**Gain**		GGTL4	0
34	Xp11.23	Yes	112	47917899…48029446	Yes	269	47917899…48186708	Yes	184	47917899…48102337	Yes	257	47935225…48192383	**Gain**		SSX5, SSX1, SSX9, SSX3	1
35	Xq13.1	Yes	185	71869375…72054837							Yes	185	71869375…72054837	**Gain**	**Yes**	DMRTC1	1

Identity of inherited CNVs found in Family 1 (7), Family 2 (8) and the gene regions which they overlap. Inherited CNVs are those which are present in either or both parents and transmitted to either or both twins. All size is in kb. SD indicates the percentage of overlap between segmental duplications and CNVs. ‘0’ means there is no overlap between CNV and segmental duplication, ‘1’ means 90–100% and ‘2’ means 50–90% overlap. Parental CNVs not transmitted to offspring were not included in Table 5–8 so the total number of CNVs present in Table 2–4 was not same as Table 5–8. The table includes genomic locations as well as individual specific break points which allow for the assessment of regions of overlap with the Database of Genomic Variants (Toronto, Ontario). SI No. = Serial number. Novel indicates a CNV which is not present in The Database of Genomic Variants (DGV).

We also analyzed genes that overlapped *de novo* CNVs (gains and losses) in order to assess their potential effect on physiology and function starting with GO ontology annotation (http://www.geneontology.org). Interestingly, the majority of genes belonged to transcription, DNA replication, transport, and cell signalling pathways, including ‘binding’ or ‘catalytic’ functions. A number of these genes are expressed in the brain, some with potential to affect neurophysiology, neurodevelopment and function and a set of them are known to show altered expression in schizophrenia (www.schizophreniaforum.org). Also of significance is the observation that the FAM19A5 protein encoded by the *FAM19A5* gene (22q13.32) belongs to the TAFA protein family which are predominantly expressed in the brain, and are postulated to function as brain-specific chemokines or neurokines, that act as regulators of immune and nervous cells [32]. This finding adds to the existing speculation about the role of the Major Histocompatability Loci (MHC) and infection in SCZ. Functional analysis of this gene and upstream regulatory elements for characteristic patterns of nucleosome occupancy changes associated with enhancers could yield novel insights into the role of this gene in psychiatric disorders. IPA analysis of gene networks of CNVs and SNPs converged on cell cycle, cellular growth and proliferation. Genes involved in genetic disorders such as hematological disease, immunological, inflammatory and developmental disorders were overrepresented. These results support the hypothesis that schizophrenia is a “developmental disorder” at the molecular level. Interestingly, a recent co-expression network analysis of microarray-based brain gene expression data revealed perturbations in developmental processes in schizophrenia [33]. However, given that these results are based on only two twin pairs, and schizophrenia is highly heterogeneous, the results on disease causations cannot be generalized. Also, we have offered other explanations for twin discordance that may involve epigenetic changes [34].

It is not surprising that genomic studies have begun to use monozygotic twins. In fact a number of them have identified copy number variations [35] and epigenetic [36]–[38] differences between them; an exception to these results is a recent study by Baranzini *et al* [39]. They studied three pairs of monozygotic twins discordant for Multiple Sclerosis (MS) and found no difference that could account for the disease causation. The results may be viewed as not surprising for a number of reasons. First, MS is known to have significant environmental components including sunlight and viruses, among others, [40] and the concordance rate in monozygotic twins is only ∼30%. Second, they assessed the CD4+ lymphocytes only that may or may not represent the causative cell type. Also, they sequenced the genome of CD4+ cells from a single pair corresponding to 21.7 and 22.5-fold coverage representing 99.6% and 99.5% of the NCBI human reference genome, which may or may not be effective. Only additional genomic and epigenomic studies on MZ twins will offer insights into the dynamics of genomic stability and change, that forms the focus of this report.

In summary, the present study adds to the recent effort in human genetics to define the phenomenon of constancy and change using inheritance and origin of genome-wide CNVs and SNPs. The results demonstrate that CNVs often result from mitosis during early development facilitated by flanking repeats. They may lead to CNV differences among different tissue and make most individuals mosaics. The described approach expands the search for disease related genetic changes, indicates the time of their occurrence and begins to interrogate the mechanisms involved.

## Materials and Methods

This research was approved by the Committee on Research Involving Human Subjects at the University of Western Ontario. The families and patients were identified, recruited and clinically assessed by Dr. Richard O'Reilly (Psychiatrist) and all participants (Figure 1) gave informed consent and provided blood and buccal cells for this research. All subjects were interviewed using the Structured Clinical Interview for DSM IV and the SCID II (for personality disorders) and their medical records collected and reviewed. Diagnoses and demographic information are listed in Table 1. DNA was extracted from the collected white blood cells using the perfect pure DNA blood kit (5prime.com) following the manufacturer's protocol. Subsequent microarray analysis was performed using the Affymetrix Genome-Wide Human SNP Array 6.0 at the London Regional Genomics Centre (LRGC) following manufacturer's protocol and stringent quality control measures. Briefly, 5 µg of genomic DNA was labelled and hybridized to Affymetrix SNP 6.0 arrays. CNVs called by both Affymetrix Genotyping Console 4.0 and Partek® Genotyping Suite™ software suites were retained for analysis. In both cases, the CNVs were identified by continuity of markers on a segment. Two CNVs that overlapped by >50% in the two methods of data analysis were given the same identity. Every measure was undertaken to avoid inclusion of false positives including correction for segmental duplications. We found evidence of CNVs associated with segmental duplications which agrees with previous studies [41]. The CNVs identified were further assessed by comparison to the Database of Genomic Variants (http://projects.tcag.ca/variation/) and annotated with gene symbols by importing the annotation file from the UCSC genome browser (NCBI36/hg 18). A CNV that was present in both members of the twin pair and not in either of their two parents was considered to be meiotic *de novo* (originated during gamete formation), while a CNV that was present in one of the two twins and not present in either parent was considered to be mitotic *de novo* (originated during development). Further, a CNV present in the SCZ affected twin only (as compared to the two parents and unaffected member of the pair or the database) was classified as “provisional *de novo* CNV” for this disease. Novel CNVs discovered in this study were validated for predicted CNVs by Real Time PCR analysis with an internal control (RNAseP gene) using TaqMan detection chemistry and the ABI Prism 7300 Sequence Detection System (Applied Biosystems, http://www.appliedbiosystems.org). The copy number of the test locus in each case was defined as 2T^−ΔΔC^ where ΔCT is the difference in threshold cycle number for the test and reference loci.

Additional CNV analysis focused on two aspects. The first deals with identification of putative repeat elements in the flanking regions of CNVs; within a 1 kb region upstream and downstream of the CNV breakpoint which could promote breakage, deletion and duplication. The identification of repeat elements was carried out using repeat masker (http://www.repeatmasker.org/). Secondly, a probable mechanism associated with sequence-specific susceptibility to CNVs was queried. This data was used to test models related to the origin of CNVs. Previously reported candidates for CNV mechanisms include Non-Allelic Homologous Recombination (NAHR), Non-Homologous End Joining (NHEJ), Fork Stalling and Template Switching (FoSTeS) and Microhomology-Mediated Break-Induced Replication (MMBIR) [42]. The second line of investigation involved functional characterization of genes by matching of the identified genes with the Schizophrenia Gene Database (http://www.schizophreniaforum.org/res/sczgene/default.asp) as well as their assessment by GO ontology (http://www.geneontology.org/). The genes identified were also subjected to IPA analysis (www.ingenuity.com) that identified the nature of gene interactions and the pathways involved.

The use of Affymetrix 6.0 Human SNP array also allowed us to assess the transmission of a total of 909622 SNPs that are contained on the array. It allowed us to identify SNPs in the twins that were not present in either of the two parents; considered to be *de novo*. The origin of the *de novo* SNPs was assumed to be parental meiosis if both twins carried the novel nucleotide. In contrast, the origin of the *de novo* SNPs was assumed to be somatic development (mitosis) if only one of the two twins carried the novel nucleotide. We were able to assign novel substitutions to different categories including their potential effect on the gene and gene product, as well as pathways that may be affected.
